# Systemic adjuvant chemotherapy for advanced malignant ocular medulloepithelioma

**DOI:** 10.1038/s41433-022-01936-4

**Published:** 2022-02-01

**Authors:** Ibrahim H. N. Sheriff, Esin K. Karaa, Tanzina Chowdhury, Irene Scheimberg, Catriona Duncan, M. Ashwin Reddy, Mandeep S. Sagoo

**Affiliations:** 1 Retinoblastoma Service, Department of Ophthalmology, The Royal London Hospital, London, UK; 2 Department of Cellular Pathology, The Royal London Hospital, London, UK; 3 Department of Paediatric Oncology, Great Ormond Street Hospital, London, UK; 4 NIHR Biomedical Research Centre for Ophthalmology, Moorfields Eye Hospital and UCL Institute of Ophthalmology, London, UK

**Keywords:** Eye cancer, Chemotherapy

## Abstract

**Background:**

Ocular medulloepithelioma (diktyoma) is a rare and potentially malignant paediatric tumour of the non-pigmented ciliary epithelium. Adjuvant chemotherapy can be given in advanced cases, but the indications and regimens remain to be defined. The aim was to identify whether adjuvant chemotherapy offers treatment benefit in advanced ocular medulloepithelioma.

**Methods:**

This was a retrospective case series of subjects referred to a single specialist ocular oncology centre for advanced ocular medulloepithelioma subsequently treated with enucleation, including those needing adjuvant systemic vincristine, etoposide and carboplatin. A case-note review was performed for included subjects meeting referral criteria. The outcomes were histopathology characteristics, recurrence, metastases and survival.

**Results:**

Between March 2010 and June 2017, four male patients (mean age 31 months) underwent enucleation for ocular medulloepithelioma. Adjuvant chemotherapy was commenced in 3 patients (75%) due to malignant histopathological features. With a mean follow-up time of 81.5 months (median 71 months, range 49–135 months) none of the patients have had recurrence, metastases or death from the tumour.

**Conclusions:**

This series is unique in reporting the management of advanced malignant ocular medulloepithelioma with adjuvant systemic vincristine, etoposide and carboplatin for advanced tumours with malignant features. This regimen appears to be safe and may be effective in preventing metastatic spread.

## Introduction

Ocular medulloepithelioma (diktyoma) is a rare tumour usually presenting in childhood arising from the nonpigmented ciliary epithelium [1], though it can rarely also arise from the optic nerve or retina [2]. First described by Verhoeff in 1904 [3], it is thought to arise from medullary epithelium in the embryo [4] and can be benign or malignant. It can be further classified into non-teratoid, made up of ciliary epithelial cells alone, or teratoid forms, which feature heteroplastic elements such as brain and muscle-like cells [5]. The underlying aetiology of ocular medulloepithelioma is uncertain in most cases. However, it has been associated with pleuropulmonary blastoma, a rare lung tumour seen in childhood related to a mutation in *DICER1*, which is involved in the regulation of gene expression [6].

Under the light microscope, ocular medulloepithelioma is characterised by a cord-like arrangement of neuroepithelial cells interspersed by undifferentiated neuroblasts which can appear similar to those seen in retinoblastoma [2]. Whilst there is some controversy over the classification of medulloepitheliomas into benign and malignant subtypes [7], it is generally thought that malignant medulloepithelioma can be distinguished from benign forms on histopathology in a number of ways. Firstly, malignant forms feature undifferentiated cells sometimes organised into Homer–Wright or Flexner–Wintersteiner rosettes as seen in retinoblastoma [8]; secondly, there is extraocular or local invasion into surrounding structures such as the cornea, choroid, sclera or optic nerve and thirdly, the tumour cells demonstrate a high mitotic rate, though this last measure is regarded as less objective [2].

Clinically, typical features of ocular medulloepithelioma include leucocoria, lens indentation and a cystic mass arising from the ciliary body, though it commonly presents with low vision or eye pain [9]. Other possible signs include cataract, glaucoma, cyclitic membrane, ectropion uveae and hyphaema [10, 11]. The often- indolent course of ocular medulloepithelioma may lead to a delay in the diagnosis as patients undergo treatment for the secondary features of the disease [12]. Ultrasound and magnetic resonance imaging (MRI) have greatly aided diagnosis, with a characteristic finding of a solid cystic mass arising from the ciliary body [13].

There are several possible treatment options for ocular medulloepithelioma, including surgery, radiotherapy and chemotherapy. Metastasis in ocular medulloepithelioma is thought to be relatively rare [14], however a series of 41 cases in which most patients underwent enucleation or partial lamellar sclerouvectomy (PLSU) reported that over a median follow up period of 24 months, 4 patients (11%) suffered recurrence and 3 patients (8%) developed systemic metastases [15]. In 2015, our group reported the use of plaque brachytherapy using ruthenium-106 as an effective treatment for selected cases of ocular medulloepithelioma; in a series of 6 cases, 80% of plaque-treated tumours regressed and there were no cases of local nor systemic recurrence [16]. A recently published case series of 6 patients treated with plaque radiotherapy concluded that whilst this method was useful for localised small to medium tumours, more advanced and larger tumours still likely require enucleation [17].

The use of systemic chemotherapy in the treatment of ocular medulloepithelioma is a topic which has generated some debate [18] but is relatively unexplored [15, 19, 20]. There are case reports in the literature detailing the use of chemotherapy mainly in the context of relapsed or metastatic disease following initial surgery [15, 19,21–24]. Chemotherapy has also been used as initial treatment as well as neo-adjuvant treatment in advanced metastatic medulloepithelioma [25, 26] as an adjuvant in the treatment of ocular medulloepithelioma of the optic nerve head [27–29], and as adjuvant therapy following exenteration for disease with extraocular extension [30].

There is therefore a need for some clarity on the role of systemic chemotherapy in the treatment of this disease. This study aims to present a series of cases in which patients with advanced ocular medulloepithelioma with malignant histopathological features were treated with adjuvant vincristine, etoposide and carboplatin with a view to assessing the safety of chemotherapy and the efficacy of this regimen in the prevention of recurrence or spread of disease.

## Subjects and methods

This was a retrospective case series of patients presenting to a tertiary referral centre with ocular signs that led to a clinical diagnosis of advanced medulloepithelioma. The Clinical Effectiveness Unit at Barts Health NHS Trust granted approval for this project (number 8960).

Patients were included if they underwent primary enucleation of the affected eye and had histologically confirmed ocular medulloepithelioma, including those who subsequently underwent adjuvant systemic chemotherapy with vincristine, etoposide and carboplatin (the ‘JOE’ regimen). Metastatic disease was screened for by bone marrow aspirate and trephine as well as lumbar puncture prior to starting treatment. The standard doses of systemic chemotherapy used for children over the age of 1 years old and greater than 10 kg in weight comprised 1.5 mg/m^2^ body surface area of vincristine, 600 mg/m^2^ of carboplatin and 300 mg/m^2^ of etoposide. The doses of each agent were adjusted for children less than 6 months of age (50% of calculated dose/m^2^) and for children 6 months to 1 year of age (75% of calculated dose/m^2^). A total of four cycles were given in the outpatient setting with approximately 21-day intervals between, provided neutrophils were ≥1 × 10^9^/l and platelets ≥100 × 10^9^/l. Side effects of this regimen include nausea, a temporary change in taste, ototoxicity (in approximately 4% of patients), bone marrow suppression requiring blood and/or platelet transfusion and altered renal function. Bloods tests were performed to monitor blood counts and renal function during and after treatment and other nephrotoxic medications were avoided whilst on treatment. If the glomerular filtration rate (GFR) was normal at the end of treatment and the patients were not also on supplements, then no further blood tests were performed. After completion of treatment, physical examinations were performed every 3-4 months for the first 3 years and then 6 monthly for 2 years, then annually thereafter.

Advanced medulloepithelioma was defined as cases that could not be treated with brachytherapy, indicating extensive tumour, widespread seeding, raised intraocular pressure or previous intraocular surgery. Outcomes recorded were clinical features, histopathology characteristics, use of systemic chemotherapy and side effect profile, recurrence, metastases and survival.

## Results

During the study period from March 2010 to June 2017, there were 4 male patients that presented with features of advanced medulloepithelioma and included in this report.

The demographic and clinical features of the series are summarised in Table 1. The mean (median, range) age at presentation was 31 months (30, 21–42). The right eye was affected in half the patients. The most common presenting symptom was red eye (2 cases) and the most common sign was raised intraocular pressure (IOP, 3 cases). Three patients had previously been diagnosed with glaucoma, two of whom had undergone glaucoma surgery and one of whom had undergone laser treatment to reduce intraocular pressure (IOP). In one case, anterior vitrectomy and vitreo-lensectomy had been performed elsewhere prior to referral.

**Table 1:** Demographic and presenting features.

Feature	No (%) *N* = 4
Referring symptoms/signs	
Red eye	2/4 (50)
Cataract	1/4 (25)
Leucocoria	1/4 (25)
Raised IOP	3/4 (75)
Mass	1/4 (25)
Pupil distortion	1/4 (25)
Previous medical history	
None	3/4 (75)
Global developmental delay	1/4 (25)
Glue ear with bilateral grommets	1/4 (25)
Sensorineural hearing loss	1/4 (25)
Vitamin D deficiency	1/4 (25)
Previous ocular history	
None	1/4 (25)
Glaucoma	3/4 (75)
Previous eye surgery	
None	2/4 (50)
Baerveldt tube insertion	2/4 (50)
Anterior vitrectomy with vitreo-lensectomy	1/4 (25)
Cyclodiode laser treatment	1/4 (25)

IOP: intraocular pressure

**Fig. 1:**
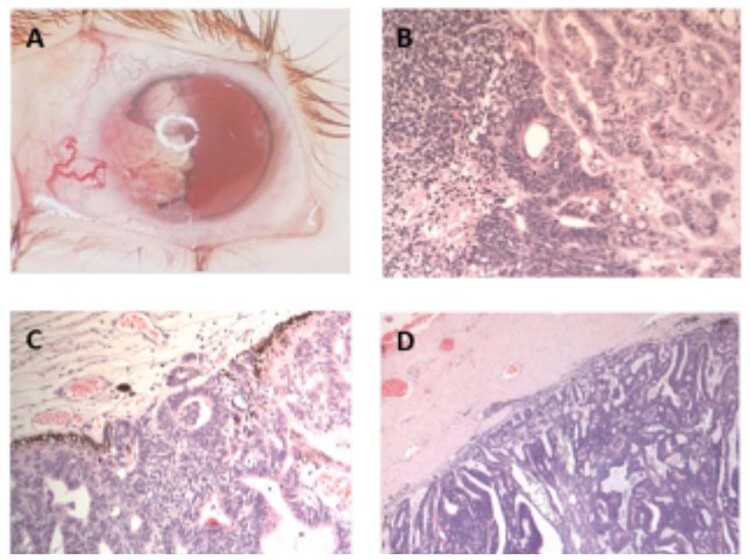
Clinical and histopathological features of malignant medulloepithelioma. **A** Characteristic white cystic mass lesion; **B** undifferentiated cell group, rosette formation and tubular structures; **C** Choroidal invasion of malignant cells; **D** Scleral invasion of malignant cells. Histopathological slides stained with Haematoxylin and Eosin.

The clinical findings are summarised in Table 2. The visual acuity in the affected eye was markedly reduced in all patients, with no light perception in 2 cases, <1.0 LogMAR at 0.5 cm in 1 case, and perception to light only in 1 case. All patients had healthy fellow eyes with a LogMAR visual acuity of 0.1 (0.1, 0.6-0.0). Mean IOP in the affected eye on presentation was 16 (12, 10–31) mmHg. The most frequent clinical feature was of a white or grey mass lesion in the anterior chamber (4 cases, Fig. 1A) with 5 (5.5, 4–6) clock hours involved. Other features included iris neovascularisation (4 cases), ectropion uveae (4 cases), cysts associated with the mass lesion (3 cases) and a cyclitic membrane (3 cases). There was also iris heterochromia in 2 cases, sectoral cataract in 2 cases and vitreous seeding in 1 case.

**Table 2:** Clinical features at presentation.

Clinical findings at presentation	No (%) *N* = 4
**Conjunctiva**	
Normal	3/4 (75)
Injection	1/4 (25)
**Cornea**	
Normal	2/4 (50)
Oedematous	2/4 (50)
**Anterior chamber**	
Shallow	1/4 (25)
Mass lesion	4/4 (100)
Cyst(s)	3/4 (75)
**Iris**	
Iris neovascularisation	4/4 (100)
Ectropion uveae	4/4 (100)
Heterochromia	2/4 (50)
Fixed pupil	1/4 (25)
Iridocorneal touch	1/4 (25)
**Lens**	
Normal	1/4 (25)
Sectoral cataract	2/4 (50)
Nuclear sclerotic cataract	0/4 (0)
Lens notch	1/4 (25)
Delaminated	1/4 (25)
**Vitreous**	
Normal	3/4 (75)
Seeds	1/4 (25)
Haemorrhage	1/4 (25)
**Fundus**	
Visible ciliary body mass	1/4 (25)
Difficult to visualise	3/4 (75)
**Colour of lesion**	
White	3/4 (75)
Grey	1/4 (25)
**Cysts**	
Absent	1/4 (25)
Present	3/4 (75)
**Cyclitic membrane**	
Absent	1/4 (25)
Present	3/4 (75)
**Quadrants involved**	
Superior-nasal	1/4 (25)
Superior-temporal	1/4 (25)
Infero-nasal	2/4 (50)
Infero-temporal	2/4 (50)
**Special investigation results**	
**B-scan**	
Mass	3/4 (75)
Enlarged ciliary body	1/4 (25)
Cysts	3/4 (75)

Three patients had a visible discrete mass on B scan/ultrasound biomicroscopy; the remaining patient had no obvious mass but was noted to have an enlarged ciliary body in the affected eye. Of those with masses seen on ultrasound, the mean sizes were transverse base 9.8 mm (9.2, 8.9–11.3) by longitudinal base 8.7 mm (8.8, 8.0–9.2) with a mean elevation of 5.1 mm (5.4, 3.9–6.0). All patients subsequently underwent enucleation with insertion of orbital implant.

The histopathological findings of enucleated eyes are summarised in Table 3. The mean medial-lateral size of the eye was 25.75 (median 24.5, range 22–32) mm; superior-inferior size was 24.5 (24, 20–30) mm and anterior-posterior size was 25.5 (23.5, 21–34) mm. The mean corneal diameter in the affected eye was 11.5 (11.5, 11-12) mm. In one patient, the tumour was not visible on the histopathological specimen macroscopically; however, it was seen microscopically. All four cases demonstrated a ‘ribbon’ like appearance on histopathology; three of the four cases also contained cells arranged in a tubular formation. All four cases showed foci with undifferentiated cells (Fig. 1B). Three cases contained cystic areas. All four cases were of the non-teratoid subtype. There were structures resembling Homer-Wright and/or Flexner-Wintersteiner rosettes in 3 of the cases. The mean optic nerve length was 6 (6, 4-8) mm and the mean optic nerve diameter was 3.25 (3, 3-4) mm. There was no optic nerve or extraocular involvement in any of the patients however there was invasion of the cornea in 2 cases, iris in 3 cases, choroid in 1 case and sclera in 1 case (Fig. 1B, C, D).

**Table 3:** Pathological characteristics of enucleated eyes.

Pathological characteristics	No (%) *N* = 4
**Teratoid vs non-teratoid subtype**	
Non-teratoid	4/4 (100)
Teratoid	0/4 (0)
**Microscopic features of cells**	
Rosettes	3/4 (75)
Fleurettes	0/4 (0)
Mitoses	3/4 (75)
Apoptosis	4/4 (100)
Cystic areas	3/4 (75)
Ribbon-like pattern of cells	4/4 (100)
Tubular arrangement of cells	3/4 (75)
**Iris invasion**	
Yes	3/4 (75)
No	1/4 (25)
**Choroidal invasion**	
Yes	1/4 (25)
No	3/4 (75)
**Retinal invasion**	
Yes	0/4 (0)
No	4/4 (100)
**Optic nerve invasion**	
Yes	0/4 (0)
No	4/4 (100)
**Corneal invasion**	
Yes	2/4 (50)
No	2/4 (50)
**Scleral invasion**	
Yes	1/4 (25)
No	3/4 (75)

Adjuvant JOE chemotherapy (vincristine, carboplatin, etoposide) was commenced in 3 of the 4 patients following enucleation (Table 4). The indication for chemotherapy was the presence of malignant features on histopathology including rosettes (3 cases), numerous mitoses (2 cases) and extension into the choroid (1 case), cornea (2 cases) or sclera (1 case). It was notable that the patient who did not receive adjuvant treatment had the smallest tumour, relatively few mitoses, no rosettes and no evidence of local spread to the choroid, cornea or sclera on histopathology.

**Table 4:** Summary of treatments and complications.

Patient	Indication for enucleation	Complications of enucleation	Adjuvant therapy (with JOE^a^ regimen)	Complications during chemotherapy	Complications after chemotherapy
1	Raised intraocular pressure; previous surgery; extensive mass	None	Yes	None	None
2	Extensive mass	None	Yes	Febrile neutropenia with positive blood cultures	Hearing impairment
3	Extensive mass	None	No	N/A	N/A
4	Raised intraocular pressure; previous surgery	Lacrimal gland prolapse	Yes	Febrile neutropenia with positive blood cultures; thrombocytopenia	Lower limb pain and paraesthesia

^a^vincristine, carboplatin, etoposide

Of those who received chemotherapy, two patients received 4 cycles of chemotherapy, and one patient received 6 cycles in total. Two patients on chemotherapy developed febrile neutropenia with positive blood cultures and recovered after appropriate treatment. After chemotherapy, one patient reported peripheral paraesthesia and one patient was noted to have hearing impairment, although it was uncertain whether this preceded treatment or was a result of it as they had preexisting hearing impairment and an undiagnosed cause of developmental delay.

At a mean follow up time to the present day of 81.5 months (median 71 months, range 49–135 months), there has been no local recurrence, metastatic spread or mortality.

## Discussion

Ocular medulloepithelioma is a rare intraocular tumour of the nonpigmented ciliary epithelium. The often-indolent nature of this tumour makes it challenging to identify and treatment for other ocular findings such as cataract or glaucoma is common without realisation of the underlying cause. In the current series, the majority of cases had been treated for glaucoma with cyclodiode laser or Baerveldt shunt, and one had undergone vitreolensectomy. One of the criteria for recommending enucleation in this tumour is that shunt surgery can act as a portal outside the eye for the seeding of malignant cells. Though metastasis is rare, the largest series of 41 ocular medulloepitheliomas demonstrated an 11% ocular recurrence rate, which was associated with a more guarded prognosis [15]. Early diagnosis and timely and effective treatment are therefore crucial to prognosis. In our previous series of cases treated by plaque radiotherapy, the rationale for this treatment was to neutralise any malignant elements in localised medulloepitheliomas. In that report tumour control rate was excellent with 80% showing signs of regression [16].

The question of optimal management of more advanced cases has received sparse attention in the literature. The use of chemotherapy is often reported as a second line treatment in relapsed disease [15]. However, chemotherapy has also been used in cases which present at a highly advanced stage with locoregional metastases, both as a neoadjuvant [23, 24] and as an adjuvant therapy [21, 25]. The use of adjuvant chemotherapy has been reported several times in the context of disease of the optic nerve head where the surgical margins were not clear of tumour [26–28]. In particular, a case of optic nerve head disease was treated in 2010 with adjuvant radiotherapy and 10 cycles of carboplatin, vincristine and etoposide, with no evidence of recurrence 2 years later [27]. Recently, there has been a case of a 5-year-old child presenting with extrascleral extension and metastases to the preauricular lymph node and parotid gland who was treated with both neoadjuvant (three cycles of vincristine, cisplatin, etoposide and cyclophosphamide) and adjuvant treatment (one further cycle of chemotherapy post enucleation); he has remained alive and disease free 9 years later [29]. Another case of a 6-year-old child with medulloepithelioma extending into the cornea and sclera was treated post exenteration with a course of chemotherapy with no recurrence at one year though the regimen used was not stated [30]. Despite these isolated cases, prior to our study there remained some controversy as to the indication for and efficacy of adjuvant chemotherapy in the treatment of ocular medulloepithelioma [31].

The rationale for the choice of vincristine, carboplatin and etoposide is in part related to their success in treating medulloepithelioma elsewhere in the body [32]. However, it also relates to the success and relative safety of this regimen as an adjuvant in treating retinoblastoma [33]. Medulloepitheliomas are known to share certain histological characteristics with retinoblastoma, such as the presence of neuroblastic cells as well as rosettes [8].

A limitation of this study is the small number of cases. As this is a rare scenario in an otherwise uncommon tumour, it is difficult to amass a larger cohort and the only way to overcome this is to develop multi centre protocols.

We have shown that a chemotherapy regimen consisting of systemic vincristine, carboplatin and etoposide appears to have been a safe adjuvant treatment following enucleation and may help to prevent recurrence and metastases. We report herein several histopathological features in which adjuvant chemotherapy may be appropriate, though a larger study with more cases will be needed to further define clear indications for chemotherapy. In this study, patients with rosette formation, numerous mitoses and local invasion were treated with adjuvant therapy and remain relapse free to the present day. There remains some discussion on how best to stratify risk and decide appropriate treatment. Previous studies have shown that adjuvant chemotherapy could help to prevent recurrence in the context of positive resection margins or clear extraocular extension, which in our experience are rare findings at presentation. Even longer follow up of existing patients as well as the experience of other major ocular oncology centres will help to elucidate optimal management for patients diagnosed with this rare tumour.

### Summary Table

#### What was known before

-Advanced ocular medulloepithelioma can carry a significant risk of morbidity including metastasis
-Plaque brachytherapy can help to regress smaller tumours and prevent recurrence in selected cases

#### What this study adds

-Adjuvant chemotherapy appears to be an effective measure to help prevent recurrence and metastasis of advanced ocular medulloepithelioma
-A regime of vincristine, carboplatin and etoposide appears to be a safe regimen in the treatment of this condition
